# BRCA1 and BRCA2 pathogenic variant carriers and endometrial cancer risk: A cohort study

**DOI:** 10.1016/j.ejca.2020.05.030

**Published:** 2020-09

**Authors:** Sarah J. Kitson, Cemsel Bafligil, Neil A.J. Ryan, Fiona Lalloo, Emma R. Woodward, Richard D. Clayton, Richard J. Edmondson, James Bolton, Emma J. Crosbie, D. Gareth Evans

**Affiliations:** a Division of Cancer Sciences, Faculty of Biology, Medicine and Health, University of Manchester, St Mary's Hospital, Manchester, UK; b Manchester Centre for Genomic Medicine, St Mary's Hospital, Manchester University NHS Foundation Trust, Manchester Academic Health Science Centre, Manchester, UK; c Department of Obstetrics and Gynaecology, Manchester University NHS Foundation Trust, Manchester Academic Health Science Centre, Manchester, UK; d Department of Histopathology, Manchester University NHS Foundation Trust, Manchester Academic Health Science Centre, Manchester, UK; e Division of Evolution and Genomic Medicine, School of Biological Sciences, Faculty of Biology, Medicine and Health, University of Manchester, Manchester Academic Health Science Centre, Manchester, UK

**Keywords:** Endometrial cancer, BRCA1, BRCA2, Risk, Serous endometrial cancer

## Abstract

**Background:**

An association between *BRCA* pathogenic variants and an increased endometrial cancer risk, specifically serous-like endometrial cancer, has been postulated but remains unproven, particularly for *BRCA2* carriers. Mechanistic evidence is lacking, and any link may be related to tamoxifen exposure or testing bias. Hysterectomy during risk-reducing bilateral salpingo-oophorectomy is, therefore, of uncertain benefit. Data from a large, prospective cohort will be informative.

**Methods:**

Data on UK *BRCA* pathogenic variant carriers were interrogated for endometrial cancer diagnoses. Standardised incidence ratios (SIRs) were calculated in four distinct cohorts using national endometrial cancer rates; either from 1/1/1980 or age 20, prospectively from date of personal pathogenic variant report, date of family pathogenic variant report or date of risk-reducing salpingo-oophorectomy. Somatic *BRCA* sequencing of 15 serous endometrial cancers was performed to detect pathogenic variants.

**Results:**

Fourteen cases of endometrial cancer were identified in 2609 women (1350 *BRCA1* and 1259 *BRCA2*), of which two were prospectively diagnosed. No significant increase in either overall or serous-like endometrial cancer risk was identified in any of the cohorts examined (SIR = 1.70, 95% confidence interval = 0.74–3.33; no cases of serous endometrial cancer diagnosed). Results were unaffected by the *BRCA* gene affected, previous breast cancer or tamoxifen use. No *BRCA* pathogenic variants were detected in any of the serous endometrial cancers tested.

**Conclusions:**

Women with a *BRCA* pathogenic variant do not appear to have a significant increased risk of all-type or serous-like endometrial cancer compared with the general population. These data provide some reassurance that hysterectomy is unlikely to be of significant benefit if performed solely as a preventive measure.

## Highlights

• Endometrial cancer risk was not increased in *BRCA1/2* pathogenic variant carriers.
• Results were unaffected by *BRCA* gene, previous breast cancer or tamoxifen use.
• No specific increase in serous-like endometrial cancer risk was identified.
• Sequencing of 15 serous endometrial tumours revealed no pathogenic *BRCA1/2* variants.

## 1 Introduction

Since the publication of a number of reports describing diagnoses of serous endometrial cancer in *BRCA1* pathogenic variant carriers of Ashkenazi Jewish heritage [[1], [2], [3]], there has been interest in a potential association between the *BRCA* pathogenic variants and an increased risk of endometrial cancer. A number of studies have sought to quantify the level of risk, although with conflicting results, with some finding evidence of an increased risk [[4], [5], [6]], particularly in *BRCA1* carriers, whilst others have found no association [7,8]. Unfortunately, the absence of a suitable control group has prevented the results of these earlier studies being reconciled in a meta-analysis [9]. From a biological perspective, should a causative relationship exist between *BRCA* pathogenic variants and endometrial cancer, it would be anticipated that the increased risk would be restricted to the serous-like histological subtype, including p53 mutant uterine carcinosaromas and mixed epithelial carcinomas [10]. This has, however, not always been observed [11,12]. It has also been postulated that any observed association may be due to the use of tamoxifen for the prevention and treatment of breast cancer rather than a consequence of a *BRCA1* or *BRCA2* pathogenic variant *per se* [13,14]. Whilst several prospective studies have examined the incidence of endometrial cancer after risk-reducing salpingo-oophorectomy (RRSO) in *BRCA1* and *BRCA2* pathogenic variant carriers compared with the general population, they have failed to consider the impact of the procedure on the rate of endometrial cancer within this specific population [12,13]. Debate, therefore, continues within the scientific and medical communities as to whether risk-reducing hysterectomy should be offered to women with *BRCA1* and *BRCA2* pathogenic variants at the time of their RRSO to reduce their subsequent endometrial cancer risk[11,15]. Limitations of the studies performed to date are that they have often been purely retrospective, have included only small numbers of *BRCA* pathogenic variant carriers, particularly those with *BRCA2* pathogenic variants, and have frequently omitted to undertake expert pathological review of the tumour tissue to ensure accurate subtyping. They have also often had short follow-up durations of only 5–6 years, which, when applied to a cohort with a median age of 40–50 years, means that they have limited power to detect endometrial cancer cases which predominately occur in older women. This study, therefore, sought to determine whether *BRCA* pathogenic variants are associated with an increased risk of endometrial cancer compared with the general population using a large, well-described cohort of *BRCA1* and *BRCA2* pathogenic variant carriers with prospective follow-ups. It also aimed to determine whether there was a particular association between *BRCA* pathogenic variants and the serous histological subtype of endometrial cancer and the impact of RRSO on this risk.

## 2 Materials and methods

A prospectively maintained database of BRCA pathogenic variant carriers at the Manchester Centre for Genomic Medicine was used to identify individuals aged >20 years for analysis. Data were collected on date of birth, personal and family pathogenic variant testing, salpingo-oophorectomy ± hysterectomy, breast, ovarian and endometrial cancer diagnosis, death and date of the last follow-up. Information on tamoxifen use was collected wherever possible. Women were eligible for the study if they had a *BRCA1* or *BRCA2* pathogenic variant identified between 01/01/1991 (the start date of the database) and 31/12/2017 and had not undergone a previous hysterectomy. Pathology reports were collated from affected individuals to determine endometrial cancer subtype, with slide review by an expert gynaecological pathologist (J.B.) where possible and TP53 immunohistochemistry in accordance with previously published protocols [16]. Follow-up data were collected through medical record review and from the National Cancer Registration and Analysis Service, for women enrolled in the Epidemiological study of Familial Breast Cancer (EMBRACE) study, a national cohort study of *BRCA1* and *2* pathogenic variant carriers and non-affected family members [17]. Women were considered in a number of distinct, but overlapping, cohorts; retrospectively assuming the follow-up started on 1/1/1980 (or age 20 years, whichever occurred later) and prospectively from the date of their family *BRCA* pathogenic variant identification (or age 20 years), from date of their personal *BRCA* pathogenic variant identification (or age 20 years) and from date of RRSO, where applicable. A nested case-control analysis was planned to evaluate the competing effect of RRSO on endometrial cancer incidence; however, no cases of endometrial cancer occurred in women who underwent RRSO. Women were censored at time of hysterectomy, diagnosis of cancer of the ovary, fallopian tube or peritoneum, death, the last follow-up or 31/12/17, whichever occurred first.

To establish whether serous endometrial cancers are associated with pathogenic variants in BRCA, we identified 15 serous endometrial cancers treated at our institution and carried out somatic BRCA sequencing. DNA was extracted from formalin-fixed paraffin embedded blocks of tumour tissue, which had been obtained at the time of hysterectomy. DNA extraction was performed using either COBAS (Cat no: 05985536190, Roche) or EZ1 (Cat no: 953034, Qiagen) extraction kits. The DNA was quantified using Qubit broad range assay and reagents. Sanger DNA sequencing for *BRCA1/2* mutations was undertaken by the Manchester Genomics Diagnostics Laboratory using their in-house developed protocol; details of which have been published elsewhere [18]. In brief, 80 ng of intact DNA was amplified using GeneRead DNAseq Targeted Exon Enrichment Breast Panel (Qiagen). PCR products were purified using Ampure XP beads and quantified on the 2200 TapeStation using D1000 High Sensitivity kit (Agilent). These were then adenylated, and adaptors were ligated using TruSeq PCR-free Library Preparation Kit according to manufacturer's protocol (Illumina). The resulting libraries were cleaned and selected for size using GeneRead (Qiagen) size selection columns, before undergoing quantification using the KAPA Library Quantification Kit (Kapa Biosystems). Each library was normalised to 0.5 nM with EB buffer (Qiagen). Samples were pooled, denatured with 0.2 N NaOH of equal volume, neutralised with 200 mM Tris of equal volume and diluted with HT1 solution (Illumina) to achieve a final 12.5pM library concentration. For sequencing, 594uL of the pooled library mix and 6 pL of 12.5pM PhiX control library were mixed and loaded on to MiSeq V.2 (Illumina). Data were processed as previously described.

Expected endometrial cancer incidence rates were calculated using age-standardised UK-specific data available from the Office for National Statistics [19] in 5 year intervals and were adjusted for local hysterectomy rates, as calculated using data from the Predicting the Risk of Cancer At Screening (PROCAS) study [20]. This was a large risk assessment study conducted in the Greater Manchester area developing breast cancer risk algorithms. The risk of endometrial cancer relative to the general population was evaluated with standardised incidence ratios (SIR), calculated as the observed number of endometrial cancer cases divided by the expected number of cases. Subgroup analyses were performed based on *BRCA1/2* pathogenic variant status, history of breast cancer and tamoxifen use and endometrial cancer histological subtype. Serous-like endometrial cancers included serous endometrial cancer, uterine carcinosarcomas with a serous epithelial component and mixed serous epithelial tumours in keeping with the findings of de Jonge et al [10]. The expected number of serous-like endometrial cancer cases was calculated assuming 10% of all endometrial cancers were of the serous-like histotype [21,22] The Byar's approximation of the exact Poisson distribution was used to calculate the 95% confidence limits using the methodology of Breslow and Day [23]. Statistical analysis was performed using MS Excel (2016).

## 3 Results

Of 2609 women, 1350 (51.7%) had a *BRCA1* pathogenic variant and 1259 (48.3%) had a *BRCA2* pathogenic variant. The median age at baseline, the last follow-up and the length of the follow-up varied according to the cohort examined (Table 1).

**Table 1:** Demographic data and follow-up duration for cohorts examined.

Cohort	Size (n)	Median age at baseline, yrs (IQR)	Median age at last follow-up, yrs (IQR)	Total follow-up, women years at risk (median)	*BRCA* pathogenic variant status (%)	Prior history of breast cancer (%)	Prior history of tamoxifen use (%)
Retrospective cohort (1/1/1980–31/12/17)	2609	20.0 (20.0–31.6)	48.8 (40.5–57.9)	59199 (23.8)	*BRCA1* 1350 (51.7%) *BRCA2* 1259 (48.3%)	Yes 1259 (48.2%) No 1350 (51.7%)	Yes 311 (11.9%) No 557 (21.3%) Unknown 1741 (66.6%)
Date of family pathogenic variant report	1811	44.1 (34.9–54.6)	49.1 (40.2–59.1)	9412 (3.5)	*BRCA1* 906 (50.0%) *BRCA2* 905 (50.0%)	Yes 907 (50.1%) No 901 (49.8%) Unknown 3 (0.2%)	Yes 260 (14.4%) No 490 (27.1%) Unknown 1061 (58.6%)
Date of personal pathogenic variant report	1617	45.1 (37.0–55.1)	49.2 (40.9–58.8)	6375 (2.4)	*BRCA1* 817 (50.5%) *BRCA2* 800 (49.5%)	Yes 847 (52.4%) No 768 (47.5%) Unknown 2 (0.1%)	Yes 258 (16.0%) No 478 (29.6%) Unknown 881 (54.4.%)
Date of RRSO	546	45.8 (40.4–52.6)	51.3 (45.4–58.9)	2865 (2.9)	*BRCA1* 274 (50.2%) *BRCA2* 272 (49.8%)	Yes 283 (51.8%) No 263 (48.2%)	Yes 103 (18.9%) No 178 (32.6%) Unknown 265 (48.5%)

IQR, interquartile range.

There were 14 cases of endometrial cancer identified; 12 occurred before the confirmation of a personal BRCA pathogenic variant (i.e. were identified retrospectively) and two cases were identified prospectively. The clinical characteristics of the endometrial cancer cases are given in Supplementary Table 1. Pathology review was possible for six of the 14 endometrial cancer cases identified, with TP53 immunohistochemistry performed in three cases to aid diagnosis. Most cases were of endometrioid subtype, with one retrospectively identified case of a mixed serous and endometrioid tumour and two cases of endometrial carcinosarcoma. Only the mixed serous tumour demonstrated diffuse p53 staining, in keeping with a mutant-like pattern. Both prospectively diagnosed endometrial cancer cases were of endometrioid subtype and occurred in index cases. There were two cases of proven synchronous ovarian and endometrial cancers, one identified prospectively and the other retrospectively, and a further suspected case within the retrospective cohort, which could not be confirmed as the original slides were not available for review. There were no cases of endometrial cancer in women who underwent RRSO.

The overall risk of endometrial cancer was not significantly increased in any of the four cohorts studied (from 1/1/1980 adjusted SIR = 1.70, 95% confidence interval [CI] = 0.74–3.33; date of family pathogenic variant report adjusted SIR = 0.89, 95% CI = 0.12–3.02; date of personal pathogenic variant report adjusted SIR = 1.21, 95% CI = 0.09–4.48; date of RRSO adjusted SIR incalculable, Table 2).

**Table 2:** Observed and expected endometrial cancer rates in BRCA pathogenic variant carriers.

Year	Expected	Observed	SIR	CI lower 95%	CI upper 95%
**1/1/1980–31/12/2017**					
1980–1984	0.28	0	0.00	0.00	0
1985–1989	0.4	0	0.00	0.00	0
1990–1994	0.61	1	1.64	0.00	13.11
1995–1999	0.82	4	4.90	0.01	31.45
2000–2004	1.08	3	2.77	0.05	14.56
2005–2009	1.35	4	2.97	0.13	13.6
2010–2014	1.3	1	0.77	0.03	3.59
2015–2017	0.44	1	2.27	0.00	23.4
Total	6.27	14	2.23	0.84	4.78
Adjusted^a^	8.22	14	1.70	0.74	3.33
Serous-like endometrial cancer	0.82	3	3.66	0.01	23.41
*BRCA1* only	3.68	7	1.9	0.47	5.05
**Date of family pathogenic variant mutation report**					
1990–1994	0	0	0	0	0
1995–1999	0.05	1	19.39	0	1420.26
2000–2004	0.2	1	5.01	0	102.28
2005–2009	0.49	0	0	0	0
2010–2014	0.65	0	0	0	0
2015–2017	0.33	0	0	0	0
Total	1.71	2	1.17	0.1	4.6
djusted^a^	2.25	2	0.89	0.12	3.02
Serous-like endometrial cancer	0.23	0	0	0	0
*BRCA1* only	0.98	1	1.02	0.01	5.73
**Date of personal pathogenic variant mutation report**					
1990–1994	0	0	0	0	0
1995–1999	0.03	1	32.35	0	3906.24
2000–2004	0.14	1	7.33	0	212.49
2005–2009	0.37	0	0	0	0
2010–2014	0.5	0	0	0	0
2015–2017	0.22	0	0	0	0
Total	1.26	2	1.59	0.06	7.55
Adjusted^a^	1.65	2	1.21	0.09	4.88
Serous-like endometrial cancer	0.17	0	0	0	0
*BRCA1* only	0.73	1	1.36	0	9.46
**Date of RRSO**					
1990–1994	0.02	0	0	0	0
1995–1999	0.04	0	0	0	0
2000–2004	0.09	0	0	0	0
2005–2009	0.19	0	0	0	0
2010–2014	0.29	0	0	0	0
2015–2017	0.13	0	0	0	0
Total	0.76	0	0	0	0
Adjusted^a^	0.99	0	0	0	0
Serous-like endometrial cancer	0.10	0	0	0	0
*BRCA1* only	0.47	0	0	0	0

CI, confidence interval; RRSO, risk-reducing salpingo-oophorectomy; SIR, standardised incidence ratio.

a Expected data adjusted for hysterectomy prevalence data.

Subgroup analyses failed to find any difference in endometrial cancer risk between women with a *BRCA1* or *BRCA2* pathogenic variant, a history of breast cancer or tamoxifen use. Neither was there a specific increase in the risk of serous-like endometrial cancer (cohort from 1/1/1980 SIR = 3.66, 95% CI = 0.01–23.41, SIR incalculable in the prospective cohorts as no cases of serous endometrial cancer diagnosed).

Furthermore, we assessed the presence of a pathogenic variant in *BRCA1/2* in first-degree relatives, of a proven carrier, who had developed endometrial cancer without previous breast or synchronous ovarian cancer. Five of seven (71%) did not carry the family variant. If endometrial cancer was associated, then more than 50% should have carried the pathogenic variant.

Of the 15 serous endometrial cancers analysed, none contained *BRCA1/2* pathogenic variants.

## 4 Discussion

This study did not find a significant increase in the incidence of endometrial cancer in women with a pathogenic variant in either the *BRCA1* or *BRCA2* genes. This finding was unaffected by the *BRCA* gene affected, a personal history of breast cancer or tamoxifen use. The study was unable to address whether RRSO reduces the risk of endometrial cancer specifically in this population, due to the lack of endometrial cancer cases in women who underwent RRSO. No specific association between *BRCA1/2* pathogenic variants and serous endometrial cancer was detected; there was neither an increased risk of serous endometrial cancer in *BRCA1/2* pathogenic variant carriers nor were pathogenic variants detected in the *BRCA1/2* genes within the tumour tissue from 15 unselected serous endometrial cancers.

These reassuring findings are consistent with those of Levine *et al.* [7], who described a relative risk of endometrial cancer of 0.75 (95% CI = 0.24–2.34, p = 0.6) in 199 Ashkenazi Jews with *BRCA1/2*, and of Lee *et al.* [11], who failed to find an increase in serous or endometrioid endometrial cancer in their moderately sized Australasian population (*BRCA1* SIR = 2.87, 95% CI = 0.59–8.43, p = 0.18, *BRCA2* SIR = 2.01, 95% CI = 0.24–7.30, p = 0.52). The largest study to date, conducted across 11 different countries, however, yielded contradictory results, noting a significantly increased risk of endometrial cancer in *BRCA1* pathogenic variant carriers and those exposed to tamoxifen [5]. Of the 4893 women studied, 3536 were *BRCA1* pathogenic variant carriers, explaining why there was no statistically significant increase in endometrial cancer risk in the *BRCA2* group, despite similar SIRs (*BRCA1* SIR = 1.91, 95% CI = 1.06–3.19, p = 0.03, *BRCA2* SIR = 1.75, 95% CI = 0.55–4.23, p = 0.2). The association between tamoxifen use and an increase in endometrial cancer incidence in BRCA pathogenic variant carriers has been confirmed in a subsequent case-control study undertaken by the same group, which found a 6.21-fold increase in risk compared with non-users (95% CI = 2.21–17.5, p = 0.0005), which the authors suggested could provide an explanation for the observed association [14]. These findings were not, however, replicated in the present study. Whilst the same authors also described a lower incidence of endometrial cancer in women who underwent oophorectomy for any reason, this has not been confirmed in other cohorts of women who have undergone specific RRSO, that is, in the absence of any tubo-ovarian disease [13]. A beneficial effect of RRSO may have been anticipated if serous endometrial cancers originate in the fallopian tube. The fact that in our cohort only one case of mixed serous and endometrioid endometrial cancer was diagnosed means that we are unable to provide any robust data to confirm or refute this hypothesis, except to state that this case occurred in a woman who had not undergone RRSO and no cases of endometrial cancer were diagnosed in the RRSO cohort.

The number of endometrial cancer cases observed in each of the cohort studies, including our own, has been small (2–17) and could well explain the difference in statistical significance of SIRs that all approximate to a value of 2. Indeed, only two cases of endometrial cancer were diagnosed in our prospective cohorts, which may indicate a testing bias in those with endometrial cancer in previously reported studies. An unbiased assessment of testing first-degree relatives with only endometrial cancer supports our premise that there is unlikely to be any substantial increase in endometrial cancer risk. It is, however, arguable that even if an SIR of 2 is validated (none of our upper confidence limits exclude this), the level of risk is insufficient to recommend hysterectomy at the time of bilateral salpingo-oophorectomy as a risk-reducing measure. Given the increased potential morbidity associated with more extensive surgery, evidence of benefit is certainly warranted to outweigh these additional risks. Whether there is a clear benefit in specific subgroups of *BRCA* pathogenic variant carriers is currently unknown; neither ours nor previously published studies have included data on body mass index (BMI) and hence have been unable to adjust for this in analyses.

The strengths of this study include the confirmation of endometrial cancer diagnoses with histological reports and contemporaneous expert pathological review of slides, although unfortunately this was not universally achievable due to the lack of availability of tumour tissue for assessment. The study also included the largest number of *BRCA2* pathogenic variant carriers to date, increasing our understanding of endometrial cancer risk in this specific population. As with previous studies, robust methodology has been employed to compare observed with national expected endometrial cancer rates, with adjustment made for local hysterectomy rates. We were able to include data on *BRCA1/2* pathogenic variant status of first-degree relatives of women who developed endometrial cancer and of unselected serous endometrial cancer cases to corroborate our findings. Our somatic BRCA testing has been shown to have high sensitivity in high-grade serous ovarian cancer [25].

The potential lack of power in our study is a limitation and one that we have attempted to address by contacting the EMBRACE study (Easton D) to ensure endometrial cancer cases have not been missed. It does mean that our ability to detect differences in endometrial cancer risk in any specific subgroups or subtype of endometrial cancer is limited. Whilst the length of the follow-up, particularly for the retrospective cohort, is a clear advantage of this work, the median age at censoring remains younger than 50 years, well below the average age of endometrial cancer diagnoses in the UK [24]. Re-analysis of the data at a later date will be performed to increase the duration of the follow-up and potentially the number of endometrial cancer diagnoses. Subgroup analyses based upon tamoxifen use was limited due to the fact that two thirds of women in the database did not have data collected on their exposure to the drug, although the vast majority of women with a pathogenic variant in a *BRCA* gene without a history of breast cancer were not known to have taken tamoxifen. The low prevalence of tamoxifen use within the cohort may well explain why no association was observed between tamoxifen use and an increase in endometrial cancer risk. Additional efforts to reduce the amount of missing data within our data set will be made to address this. Data were unfortunately not routinely collected on hormone replacement therapy (HRT) use by women who underwent RRSO and so the impact of this on subsequent endometrial cancer risk could not be assessed. Whilst oestrogen-only HRT may be associated with a lower rate of subsequent breast cancer [25,26], this must be balanced against the impact this could have on the risk of malignant changes within the endometrium. Whilst every attempt was made to undertake histological review of all endometrial cancer cases occurring within the cohort, this was unfortunately not possible for eight cases where slides were unavailable. Four of these cases were also operated on at another hospital and, as it was not possible to retrieve the pathology reports for these tumours, this may impact upon the results of our subgroup analysis of serous-like endometrial cancers.

## 5 Conclusion

In conclusion, *BRCA1* and *BRCA2* pathogenic variant carriers do not appear to be at a significant increased risk of endometrial cancer compared with the general population. Neither does there appear to be a specific association between *BRCA1/2* pathogenic variants and serous endometrial cancer. Women and clinicians should be reassured that hysterectomy at the time of bilateral salpingo-oophorectomy is unlikely to be of benefit if performed solely for the purpose of trying to reduce subsequent endometrial cancer risk.

## Ethics approval and consent for publication

All women consented to the inclusion of their personal information in a prospective database held at the Manchester Centre for Genomic Medicine (The study was approved by the Central Manchester Research Ethics Committee (10/H1008/24 and 11/H1003/3), the review of their clinical records and cancer registry data for information on subsequent cancer diagnoses and the publication of any studies undertaken using their anonymised data. The endometrial tumour tissue used for *BRCA1/2* sequencing was donated by women enrolled into other, unrelated, studies at our institute [27] (Proportion of Endometrial Tumours Associated Lynch Syndrome (PETALS) study North West Research Ethics Committee reference 15/NW/0733, Cancer Research UK clinical trial database, ref-13595). The study was performed in accordance with the Declaration of Helsinki.

## Data availability

The data that support the findings of this study are available from the corresponding author upon request.

## Funding

S.J.K. reports being a 10.13039/501100000272National Institute for Health Research (NIHR) Academic Clinical Lecturer. N.A.J.R. reports being a Medical Research Council Doctoral Research Fellow (MR/M018431/1). E.J.C., E.R.W., C.B. and D.G.E. are supported through the NIHR 10.13039/100014653Manchester Biomedical Research Centre (IS-BRC1215-20007), and D.G.E. reports being an 10.13039/100006662NIHR Senior Investigator (NF–SI-0513-10076).

## Role of the funding source

The funding source had no role in the design of this study, its execution, analysis, interpretation of the results or decision to submit the results.

## Author contributions

E.J.C. and D.G.E. contributed in designing the study and supervising its execution. F.L., E.R.W., R.D.C., R.J.E. and D.G.E. contributed in data collection. S.J.K. performed the analyses and drafted the manuscript. C.B. and N.A.J.R. contributed to data collection and analysis. J.B. provided pathological review of tumour slides. All authors contributed to the final manuscript.

## Conflict of interest statement

The authors report no conflicts of interest.
