# Supplementary material for: BRCA1 and BRCA2 pathogenic variant carriers and endometrial cancer risk: A cohort study
Source: Eur J Cancer. 2020 Sep;136:169–75. doi: 10.1016/j.ejca.2020.05.030 (PMC7441309; doi:10.1016/j.ejca.2020.05.030)
Supplement: Multimedia component 1 [file mmc1.docx]

| **Pt** | **Prospective/retrospective endometrial cancer diagnosis** | **Age at start of follow-up** | ***BRCA1/2*** | **History of breast cancer?** | **History of prior tamoxifen use?** | **Previous RSSO?** | **Time from start of follow-up to diagnosis (yrs)** | **Histology** | **FIGO (2009) stage** | **Status** | **Pathology review?** | **Additional details** |
| --- | --- | --- | --- | --- | --- | --- | --- | --- | --- | --- | --- | --- |
| 1 | Prospective | 54.5 | *BRCA1* | Yes | No | No | 2.3 | Endometrioid | 1b | Dead | Yes | Synchronous diagnosis of stage 3c ovarian cancer. Death related to ovarian cancer |
| 2 | Prospective | 50.2 | *BRCA2* | Yes | No | No | 3.0 | Endometrioid | 1b | Alive | No |  |
| 3 | Retrospective | 42.0 | *BRCA1* | No | No | No | 13.4 | Adenocarcinoma | 3 | Dead | Yes | Morphology in keeping with endometrial origin. No subtype information available |
| 4 | Retrospective | 40.4 | *BRCA1* | Yes | Unknown | No | 25.3 | Unknown | Unknown | Dead | No | Unable to locate histology report |
| 5 | Retrospective | 36.5 | *BRCA2* | Yes | Yes | No | 16.4 | Endometrioid | 1a | Alive | No |  |
| 6 | Retrospective | 37.8 | *BRCA2* | No | No | No | 17.7 | Endometrioid | 2 | Alive | No |  |
| 7 | Retrospective | 41.9 | *BRCA1* | Yes | Unknown | No | 16.0 | Endometrioid | 1a | Alive | Yes |  |
| 8 | Retrospective | 41.5 | *BRCA2* | Yes | Yes | No | 27.7 | Endometrioid | Unknown | Alive | No | Unable to confirm stage |
| 9 | Retrospective | 39.9 | *BRCA2* | Yes | Yes | No | 33.3 | Mixed serous and endometrioid | 1a | Alive | Yes |  |
| 10 | Retrospective | 40.5 | *BRCA1* | No | No | No | 29.9 | Unknown | Unknown | Deceased | No | Unable to locate histology report. Simulataneous ovarian cancer diagnosis. Death related to ovarian cancer |
| 11 | Retrospective | 52.7 | *BRCA2* | Yes | Unknown | No | 23.6 | Carcinosarcoma | 3c | Deceased | Yes | Death related to breast cancer |
| 12 | Retrospective | 37.0 | *BRCA2* | No | No | No | 23.4 | Unknown | Unknown | Alive | No | Unable to locate histology report |
| 13 | Retrospective | 23.6 | *BRCA1* | No | No | No | 26.0 | Endometrioid | Unknown | Deceased | Yes | Tubal tumour deposit reported on original report, but slides unavailable for review |
| 14 | Retrospective | 20.0 | *BRCA1* | Yes | Unknown | No | 31.6 | Carcinosarcoma | Unknown | Alive | No | Unable to locate slides for expert review |
